# Anxiolytic and antidepressant-like effects of essential oil from the fruits of *Piper nigrum* Linn. (Black pepper) in mice: involvement of serotonergic but not GABAergic transmission system

**DOI:** 10.1016/j.heliyon.2021.e06884

**Published:** 2021-04-25

**Authors:** Sourav Ghosh, Arvind Kumar, Neetu Sachan, Phool Chandra

**Affiliations:** a School of Pharmaceutical Sciences, IFTM University, Lodhipur Rajput, Delhi Road (NH-24), Moradabad, 244 102, UP, India; b Department of Pharmaceutical Chemistry, S. D. College of Pharmacy & Vocational Studies, Bhopa Road, Muzaffarnagar, 251001, UP, India

**Keywords:** Anxiolytic, Antidepressant, Essential oil, *Piper nigrum*, Serotonergic transmission

## Abstract

In this study, the anxiolytic activity of *Piper nigrum* essential oil (PNEO) was evaluated in the elevated plus maze (EPM) and the antidepressant-like effect was evaluated through tail suspension test (TST) in mice. Flumazenil, a competitive inhibitor of GABA_A_ receptor in the benzodiazepine site and WAY-100635 maleate salt, a 5-HT_1A_ receptor antagonist were used to find out the possible mechanism(s) of action of PNEO. To exclude the false-positive results due to the enhancement of the locomotor activity, the animals were submitted to open field test (OFT). We also measured monoamines levels of the mice brain after acute PNEO treatment. The data obtained from the study suggest that the anxiolytics and antidepressant-like effect of PNEO have observed in EPM and TST respectively in a dose-dependent manner after oral acute and repetitive treatment. WAY-100635, but not flumazenil was able to reverse the effect of PNEO in EPM and TST both, indicating the possible involvement of 5-HT_1A_ receptor. The neurochemical analysis showed no alteration in monoamine levels in mice brains. Furthermore, no locomotor impairment or sign of toxicity or changes in body weight or abnormalities in the biochemical parameters, except for a significant decrease in total cholesterol level was observed after treatment with PNEO. The findings suggest that *Piper nigrum* EO possesses a dual anxiolytic and antidepressant-like effect through the possible involvement of serotonergic transmission.

## 1 Introduction

Anxiety is the most frequent neuropsychiatric disorder that affects anyone at all age groups. More than one-eighth of the global population suffer from this disorder at some stages during their life (Grundmann et al., 2009). The first-line treatment of these disorders commonly employs barbiturates, benzodiazepines and azapirone but imposes some serious adverse effects including motor dysfunction, deficits in attention, sedation, convulsion and sexual disability (Grundmann et al., 2009). Moreover, the degree of improvement in patients is quite dissatisfying and chronic treatment with these drugs produces tolerance (Doukkali et al., 2015). Similarly, depressive disorder is another increasingly prevalent neuropsychiatric disorder. According to WHO near about 4.4 % of the global population are living with depression. Depression will soon become a major cause of disability and morbidity after cardiac diseases. Furthermore, coexistence of anxiety and depression are often associated together and seen in same individuals. Recent evidences suggested that antidepressant drugs are more effective in the treatment of anxiety disorders via two major biochemical pathways namely, central serotonergic system and GABA-BZD receptor complex (Suranyi-Cadotte et al., 1990). It has been reported that available antidepressants are only effective for few patients and often cause adverse effects including cardiotoxicity, sleep disorders and weight gain that making their use limited (Deng et al., 2015; Pesarico et al., 2014). Therefore, there is an urgent requirement for the development of new psychopharmacological agents having a rapid onset of action along with minor adverse effects. In this aspect, aromatic medicinal plants have been increasing their importance as the rich diversity in bioactive agents that have led to the development of important therapeutic agents related to these disorders. Essential oils (EOs) are compound mixture of low molecular weight lipophilic volatile components that are water insoluble and permeable to the blood –brain barrier (Pavela and Benelli, 2016). Aromatherapy with essential oils (EOs) extracted from these plants is a long traditional practice in folk, Chinese and traditional medicine system (Dobetsberger and Buchbauer, 2011). Many of them have been reported to treat numerous neurological disorders including anxiety and depression (Diniz et al., 2019; Oliveira Júnior et al., 2018).

*Piper nigrum* Linn. belongs to the Piperaceae family, native to western ghats of India and tropical region of southeast Asia. The dried unripe fruit, ‘black pepper’ utilized as a spice in all appetizing cooking worldwide and shows an extensive range of pharmacological activities (Takooree et al., 2019). The essential oil from the fruits of *Piper nigrum* has a musky, spicy and warm aroma and is reported to have several biological activities including antioxidant, anti-inflammatory and anti-nociceptive (Jeena et al., 2014). In traditional medicine, black pepper has been used to treat epilepsy in China. Also, fruits from *piper nigrum* (black pepper) have been used as a brain tonic in traditional Middle Eastern medicine (Majeed and Prakash, 2000). As far as we know, there are no scientific studies that show the possible neuropharmacological effects of EOs from fruits from *piper nigrum*. However, some studies suggested that EOs from other piper spp. exhibited anxiolytics, sedative, muscle relaxant, antipsychotic and anticonvulsant effects (Oyemitan et al., 2015; Tankam and Ito, 2013).

Therefore, this study was undertaken to explore the anxiolytics and antidepressant-like effects of acute and repetitive treatment with *Piper nigrum* essential oil (PNEO) using open field test (OFT), elevated plus maze (EPM) and tail suspension test (TST) in mice. Furthermore, the possible mechanism(s) of action participated in the anxiolytics and antidepressant-like effects of acute treatment with PNEO in EPM and TST along with neurochemical study was investigated. To evaluate the long term effect of the PNEO, periodic changes in body weight and biochemical parameters were also monitored.

## 2 Materials and methods

### 2.1 Extraction of essential oil

The fruits of *Piper nigrum* were collected from the local market of muzaffarnagar district, Uttar Pradesh, India. The collected fruits were identified by Dr. Sunita Garg, Emeritus Scientist, CSIR- National Institute of Science Communication & International resources, Pusa, New Delhi-110012 India. The voucher specimen (NISCAIR/RHMD/Consult/2019/3427-28-2) have been deposited in the Raw Materials Herbarium & Museum (RHMD), Delhi, India. For the extraction of essential oils from fruits of *Piper nigrum*, powdered material was hydrodistilled for 4 h in a Clevenger type of apparatus to yield 1.4% transparent essential oil (PNEO) with a characteristic odor. The oil was dried over anhydrous Na_2_SO_4_ and placed in a refrigerator at 4 °C before the chemical and neuropharmacological analysis.

### 2.2 Chemical analysis of essential oil

Chemical constituents present in PNEO were analyzed by Gas chromatography combined with mass-spectrometer (Shimadzu, Japan). The temperature of the fused silica capillary column was set to 70 °C for an initial hold period of 2m, then to 250 °C for a final hold period of 3m, splitless injection mode was used. Helium was taken as a carrier gas at a flow rate of 10.4 ml/m and MS detector was used in complete scan mode with EI ionization at 70eV. The components present in PNEO were identified by integrating a computer library search based on the similarity of fragmentation patterns of mass spectrum with those documented in NIST library, as well as visual interpretation of mass spectra and retention indices calculated using the n-alkane homologous series as a reference.

### 2.3 Animals and ethical guidelines

Adult male albino Swiss mice (25–30g) were purchased from the National Institute of Biologicals, Noida, Uttar Pradesh, India. Animals were housed with proper food and water in the animal house of S. D. College of Pharmacy and Vocational Studies (S.D.C.O.P & VS) under a 12-12h light-dark cycle at regulated room temperature and humidity. All the conducted animal experiments were approved by the Institutional Animal Ethics Committee (IAEC) of S.D.C.O.P & VS, Department of Pharmacology (Approval No. SDCOP&VS/AH/CPCSEA/02/08).

### 2.4 Drug and chemicals

Diazepam (DZP, Svizzera Healthcare, India) and Buspirone HCl (BSP, Sigma-Aldrich, U.S.A) was used as reference drugs. Flumazenil (FLU, Sigma-Aldrich, U.S.A), a competitive inhibitor of GABA_A_ receptor in the benzodiazepine site and WAY-100635 maleate salt (WAY, Sigma-Aldrich, U.S.A) a 5-HT_1A_ receptor antagonist was used to find out the possible mechanism(s) of action. PNEO was dissolved in 0.01% (v/v) Tween 80 (Central Drug House, India) in saline and treated orally (p.o.). For the intraperitoneal treatments, the reference drugs and other agents were given by dissolving in normal saline (0.9 %w/v NaCl). All samples were freshly prepared before the behavioral testing and treated at 0.1 ml/10 g of body weight (b/w), except Flumazenil, which was treated at 0.2 ml/10 g of b/w.

### 2.5 Experimental designing and treatments

**Figure 1:**
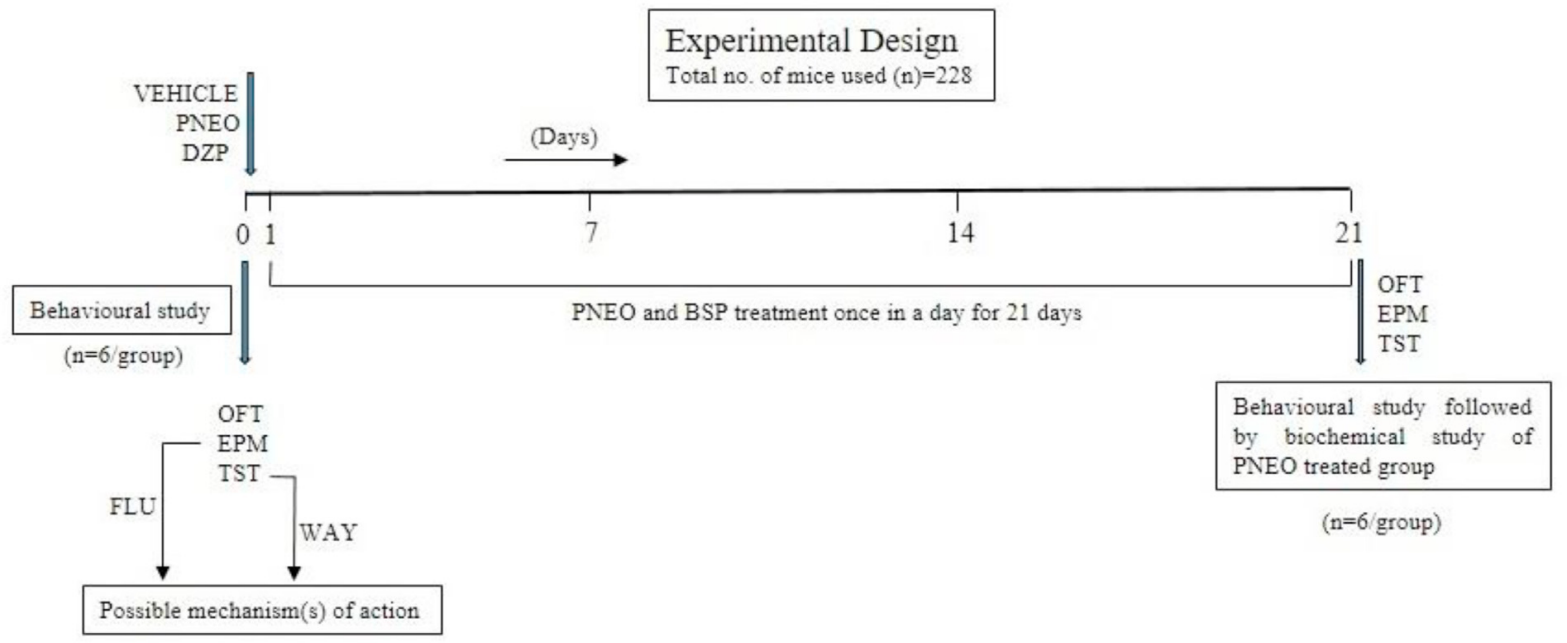
Schematic illustration of the animal experimental design. In the acute study, vehicle (control) and *Piper nigrum* EO (PNEO 5, 10 and 50 mg/kg) were orally administered. The reference drug, Diazepam (DZP 1 mg/kg) was administered intraperitoneally in the behavioural studies like Open field test (OFT), Elevated plus maze (EPM) and Tail suspension test (TST). To investigate the possible mechanism of action the antagonist flumazenil (FLU 3 mg/kg) and WAY-100635 (WAY 1 mg/kg) were administered intraperitoneally 15 min before oral treatment. In repetitive study, Control, PNEO (5, 10 and 50 mg/kg, p.o) and reference drug Buspirone (BSP 10 mg/kg, i.p) were administered once in a day for 21 days. On the last day, behavioural and biochemical study were performed.

Since there was no widely accepted dosage of *P.nigrum* EO for neuropharmacological activity, the lowest dose of 5 mg/kg of b/w was chosen, as it had previously shown to have a significant anxiolytic and antidepressant-like efffect in a pilot study. All experiments were performed using a total number of 228 mice. To evaluate the anxiolytic and antidepressant-like effect of the acute treatment of PNEO, the mice (n = 6 per group) were randomly divided into five study groups: vehicle (CON), DZP (1 mg/kg), PNEO (5 mg/kg), PNEO (10 mg/kg), PNEO (50 mg/kg) and for the treatment with repeated dose, mice were treated with vehicle control, PNEO and reference drug once in a day for 21 days(WHO, 1993). In this case, mice (n = 6 per group) were also randomly divided into five study groups as follows vehicle (CON), BSP (10 mg/kg), PNEO (5 mg/kg), PNEO (10 mg/kg), PNEO (50 mg/kg). Despite the consistent effect of diazepam after acute treatment, we have selected buspirone (a 5-HT_1A_ receptor antagonist with a slow onset of action and inconclusive acute effect) as a reference drug in repetitive treatment due to the cognition impairment was associated with long term use of diazepam in mice (Barrett and Vanover, 1993; Salomons et al., 2012). To evaluate the possible mechanism(s) of action behind the anxiolytic and antidepressant-like effect of the acute treatment of PNEO, FLU (a competitive inhibitor of GABA_A_ receptor in the benzodiazepine site) and WAY (a 5-HT_1A_ receptor antagonist) were pre-treated. For this purpose, mice (n = 6 per group) were also randomly divided into six study groups: vehicle (CON), PNEO (10 mg/kg), FLU (3 mg/kg), FLU (3 mg/kg) + PNEO (10 mg/kg), WAY (1 mg/kg), WAY (1 mg/kg) + PNEO (10 mg/kg). The experimental design is represented in Figure 1.

### 2.6 Behavioural study

#### 2.6.1 Open field test (OFT)

The open field apparatus was made of acrylic glass (30 × 30 × 15 cm) that was divided into nine squares. The study procedure entails putting an animal in an uncertain situation from which escape is impossible due to surrounding walls. This apparatus was used to study animal exploratory behaviour and anxiety activity. During a 5-m period, the number of squares crossed with the four paws by each mouse, as well as the number of grooming and rearing activities, were recorded (Nogueira Neto et al., 2013).

#### 2.6.2 Elevated plus maze (EPM) test

EPM is used to examine anxiolytic activity. The apparatus consists of two open and closed arms. In a face to face position and arms were elevated at a certain height of 40 cm. All treatments were made 30 min before being placed on the animal on the central base of EPM. For a period of 5m, the number of entries in the open arm (NEOA) and time of permanence in the open arm (TPOA) were recorded (Oliveira Júnior et al., 2018).

#### 2.6.3 Tail suspension test (TST)

TST was employed to assess the antidepressant-like effect of *Piper nigrum* essential oil in mice. Animals were suspended 30 cm away from the floor by the use of adhesive tape. The tape was attached around 1cm from the tip of the animal's tail. All treatments were made 30 min before the experimental procedure. Immobility time (completely immobile in suspending position) was recorded for 6 m (Diniz et al., 2019).

### 2.7 Mechanism(s) of action involved in the anxiolytic and antidepressant-like effect of PNEO

Mice were pre-treated (15 m before) with flumazenil or WAY-100635 to investigate whether GABA-ergic and serotonergic transmission were involved in the effect of PNEO observed in EPM and TST. Animals were evaluated 30 min after acute treatment with PNEO or vehicle (control).

### 2.8 Neurochemical analysis

The mice were treated with an acute dose of PNEO of 5, 10 or 50 mg/kg before being sacrificed right after the tail suspension test., The brain tissue was weighed and homogenized manually with ice-cold 0.1 M perchloric acid, centrifuged at 10,000 rpm for 20 m and stored at -80 °C until the determination of monoamines level. The level of monoamines (dopamine and serotonin) and their metabolites (3, 4-dihydroxyphenylacetic acid, homovanillic acid and 5-hydroxy indole acetic acid) were measured by reverse-phase high-performance liquid chromatography (HPLC) using a system (Water 515, Milford, Massachusetts, U.S.A) With an electrochemical detector. 20 μl supernatant homogenate was loaded into a sample injector and the mobile phase (50 mM Sodium citrate at pH 3.5, 0.3 mM sodium edetate, 1.8 mM dibutylamine and 4% methanol) was delivered at a constant rate of 0.35 ml/m. Every sample had a 15-minute runtime, and all monoamines and their metabolites were measured in nanograms per gram (ng/g) of fresh tissue weight (Costa et al., 2013; Li et al., 2016).

### 2.9 Toxicity study and biochemical analysis

Mice were given a regular dosage of 5, 10, or 50 mg/kg PNEO in EPM and FST for 21 days and were observed daily for common signs of toxicity like irritability, reaction to contact, gripping the tail, twisting, writhing, grip strength, ataxia, tremor, convulsion, lacrimation, piloerection and respiratory frequency. The mice were periodically weighed on days 1, 7, 14 and 21 to evaluate changes in body weight. After the accomplishing of the general behavioral toxicity study, blood was collected for biochemical evaluation. Biochemical analysis was performed by centrifuging the collected blood (without anticoagulant) at 4 °C in 3000 rpm for 10 m and the separated serum was stored at -20 °C until further use. Biochemical parameters like aspartate aminotransferase (AST), alanine aminotransferase (ALT), alkaline phosphatase (ALP), triglycerides, total cholesterol, HDL, LDL, glucose, creatinine and urea were analyzed by different test kits (ARKRAY Healthcare Pvt. Ltd, India).

### 2.10 Statistical analysis

All data were expressed as mean ± standard error of the mean (SEM). Statistical analysis was performed using one-way ANOVA followed by Dunnett's test comparison between control and all treated groups implementing Dunnett's multiple comparison test by the use of GraphPad Prism software (version 5).In all experiments, differences in data were considered significant at a 95% confidence interval.

## 3 Results

### 3.1 Chemical compositions of PNEO

The active constituents present in the essential oil of *Piper nigrum* were identified using gas chromatography-mass spectrometry (GC-MS), and thirty-three compounds were identified, accounting for 98.33% of the total composition (Table 1). Monoterpene hydrocarbons dominated the PNEO composition, accounting for 73.40% of the total. The most abundant components were limonene (25.34%), sabinene (22.86 %), and β-pinene (10.43%). Other minor components of this group were α-pinene (7.84%), thujene (2.76%) and α-phellandrene (1.12%). Sesquiterpenes hydrocarbons (23.51%) made a small contribution to the PNEO, with caryophyllene (13.38%), bisabolene (3.98%) and α-copaene (2.83%) as the most representative compounds.

**Table 1:** Chemical composition of essential oil of *Piper nigrum* (PNEO).

No.	Component	Formula	RI	% of abundance	Identification
1.	Thujene	C_10_H_16_	902	2.76	MS, RI, RT
2.	α-pinene	C_10_H_16_	936	7.84	MS, RI, RT
3.	Camphene	C_10_H_16_	943	0.22	MS, RI, RT
4.	Sabinene	C_10_H_16_	967	22.86	MS, RI, RT
5.	β-pinene	C_10_H_16_	972	10.43	MS, RI, RT
6.	Myrecene	C_10_H_16_	992	1.68	MS, RI
7.	α-phellaldrene	C_10_H_16_	1003	1.12	MS, RI
8.	δ-3-carene	C_10_H_16_	1013	0.49	MS, RI
9.	α -Terpinene	C_10_H_16_	1018	0.11	MS, RI
10.	Limonene	C_10_H_16_	1030	25.34	MS, RI, RT
11.	β-Ocimene	C_10_H_16_	1037	0.15	MS, RI
12.	β-cymene	C_10_H_14_	1042	0.22	MS, RI
13.	γ – Terpinene	C_10_H_16_	1062	0.18	MS, RI
14.	Limonene oxide –cis	C_10_H_16_O	1135	0.12	MS, RI
15.	Terpinene-4-ol	C_10_H_18_O	1137	0.56	MS, RI
16.	Limonene oxide –trans	C_10_H_16_O	1139	0.11	MS, RI
17.	α-Terpineol	C_10_H_18_O	1186	0.18	MS, RI
18.	α-Cubebene	C_15_H_24_	1344	0.28	MS, RI
19.	α -Copaene	C_15_H_24_	1378	2.83	MS, RI
20.	α-Bergamotene	C_15_H_24_	1424	0.39	MS, RI
21.	β -Copaene	C_15_H_24_	1430	0.17	MS, RI
22.	Zingiberene	C_15_H_24_	1451	0.56	MS, RI
23.	Humulene	C_15_H_24_	1458	0.38	MS, RI
24	β-Farnesene	C_15_H_24_	1461	1.12	MS, RI
25.	Cubebol	C_15_H_26_O	1472	0.14	MS, RI
26.	Caryophyllene	C_15_H_24_	1484	13.38	MS, RI
27.	α-Guainene	C_15_H_24_	1490	0.32	MS, RI
28.	Germacrene-D	C_15_H_24_	1515	0.10	MS, RI
29.	Bisabolene	C_15_H_24_	1518	3.98	MS, RI
30.	Viridiflorol	C_15_H_26_O	1530	0.23	MS, RI
31.	Caryophyllene oxide	C_15_H_24_O	1576	0.14	MS, RI
32.	α -Cadinol	C_15_H_26_O	1580	0.15	MS, RI
33.	α-Bisbolol	C_15_H_26_O	1616	0.11	MS, RI
	**Total identified**			98.33	
	Monoterpene hydrocarbons			73.40	
	Oxygenated monoterpenes			0.97	
	Sesquiterpene hydrocarbons			23.51	
	Oxygenated sesquiterpenes			0.45	

RI: Identification based on retention indices corresponding to of n-alkanes homologous series; RT: Identification based on retention time of compounds, MS: Identification based on comparison of mass spectra.

### 3.2 Behavioural study

#### 3.2.1 Effects of PNEO in the open field test (OFT)

**Figure 2:**
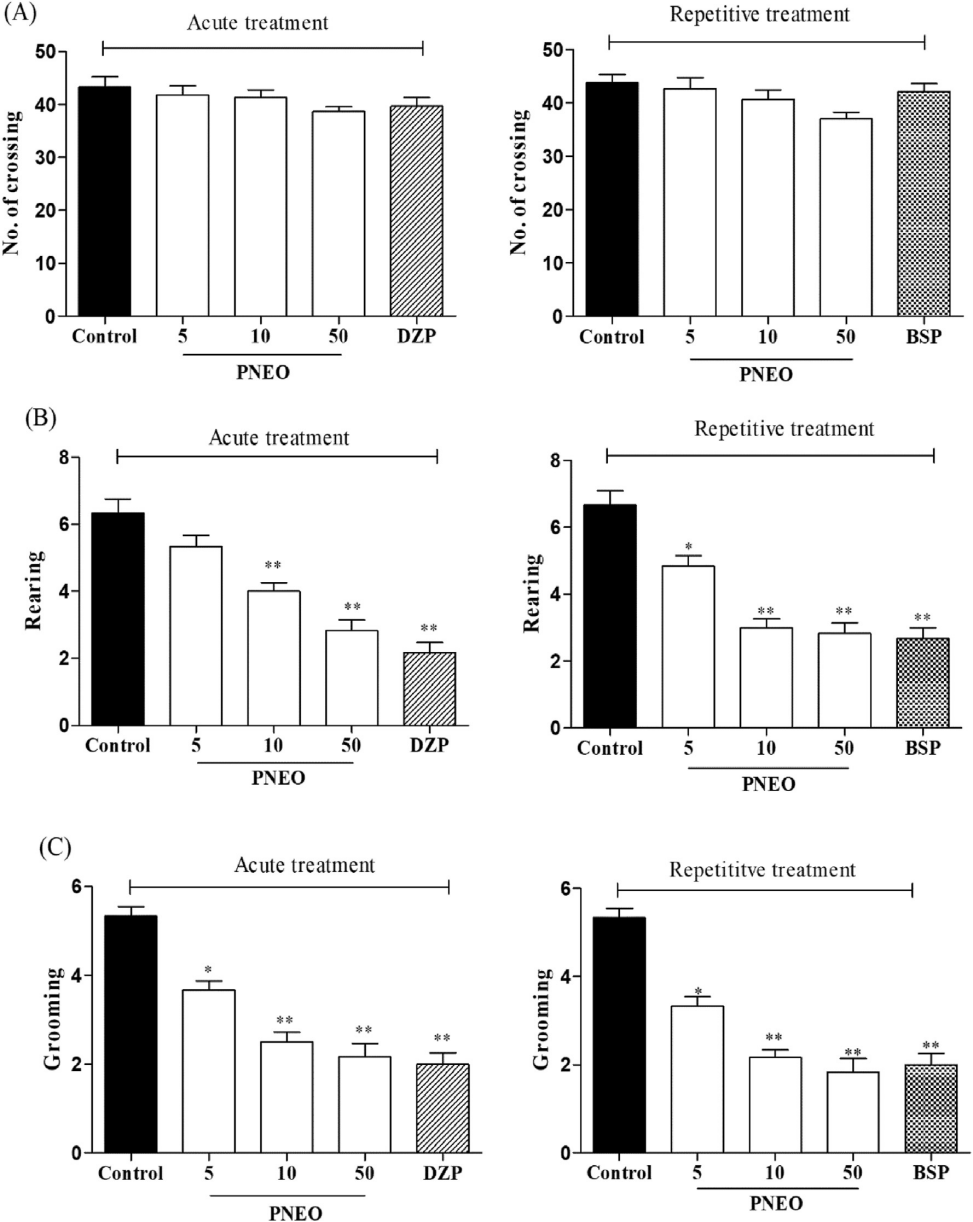
The effect of acute or repetitive treatments essential oil of *P.nigrum* (PNEO 5, 10 and 50 mg/kg, p.o.) on (A) no. of crossing (B) rearing (C) grooming in the open-field test (OFT). DZP: diazepam, 1 mg/kg (i.p.); BSP: buspirone, 10 mg/kg (i.p.). Data are represented as mean ± SEM (n = 6) and the values obtained by comparison with control group using One-way ANOVA followed by Dunnett's test, where ∗ and ∗∗ indicates *p < 0.05* and *p < 0.01* respectively.

The OFT was carried out to access the exploratory behavior of the PNEO in mice. As represented in Figure 2(A), acute and repeated treatment of PNEO, diazepam and buspirone treatments had no effect on the number of crossings (overall locomotor activity)., but the number of rearing behavior significantly reduced by acute (10, 50 mg/kg) and repetitive treatment (5, 10, 50 mg/kg) of PNEO. Moreover, grooming behavior is also reduced significantly by acute and repetitive treatment of PNEO at all doses. Similarly, diazepam (single dose) and buspirone (repetitive dose) administration, significantly decreased the number of rearing and grooming behaviours as shown in Figure 2 (B and C).

#### 3.2.2 Effects of PNEO in the elevated plus maze (EPM) test

**Figure 3:**
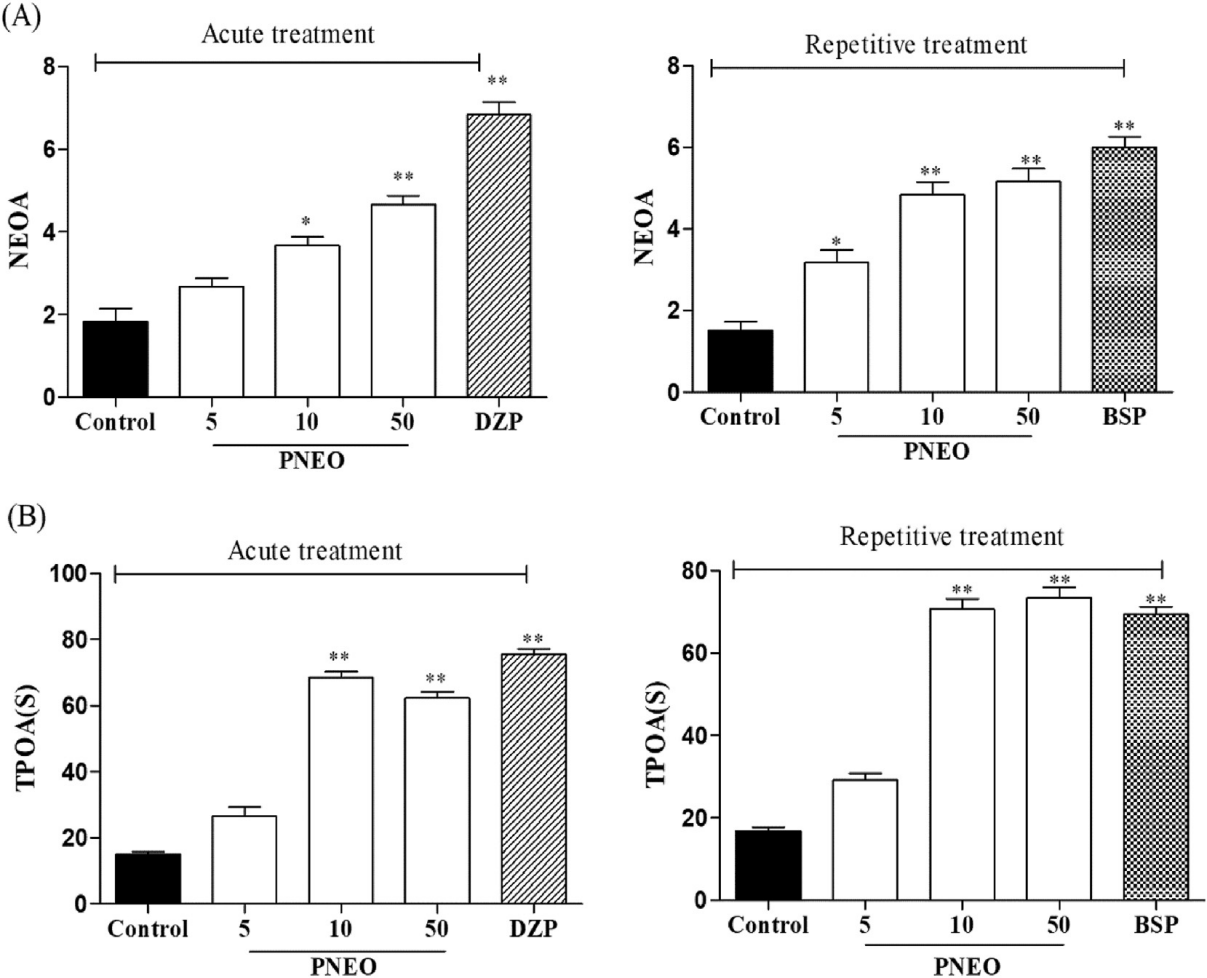
The effect of acute or repetitive treatments essential oil of *P. nigrum* (PNEO 5. 10 and 50 mg/kg, p.o.) on (A) NEOA: Number of entries in the open arms. TPOA: Time of permanence in the open arms in the elevated plus maze (EPM). DZP: diazepam, 1 mg/kg (i.p.); BSP: buspirone, 10 mg/kg (i.p.). Data are represented as mean ± SEM (n = 6) and the values obtained by comparison with control group using One-way ANOVA followed by Dunnett's test, where ∗ and ∗∗ indicates *p < 0.05* and *p < 0.01* respectively.

The EPM was used to investigate the anxiolytic effect of the PNEO after acute and repetitive treatment. The no. of open arm entries (NEOA) and the period of permanence in the open arm (TPOA) increased significantly after acute administration of PNEO 10, PNEO 50, and diazepam when compared with the control group (Figure 3A and 3B, left panel). Repeated treatment with all doses of PNEO and the reference drug, buspirone for 21 days also increased the NEOA and TPOA significantly (Figure 3A and 3B, right panel).

#### 3.2.3 Effects of PNEO in the tail suspension test (TST)

**Figure 4:**
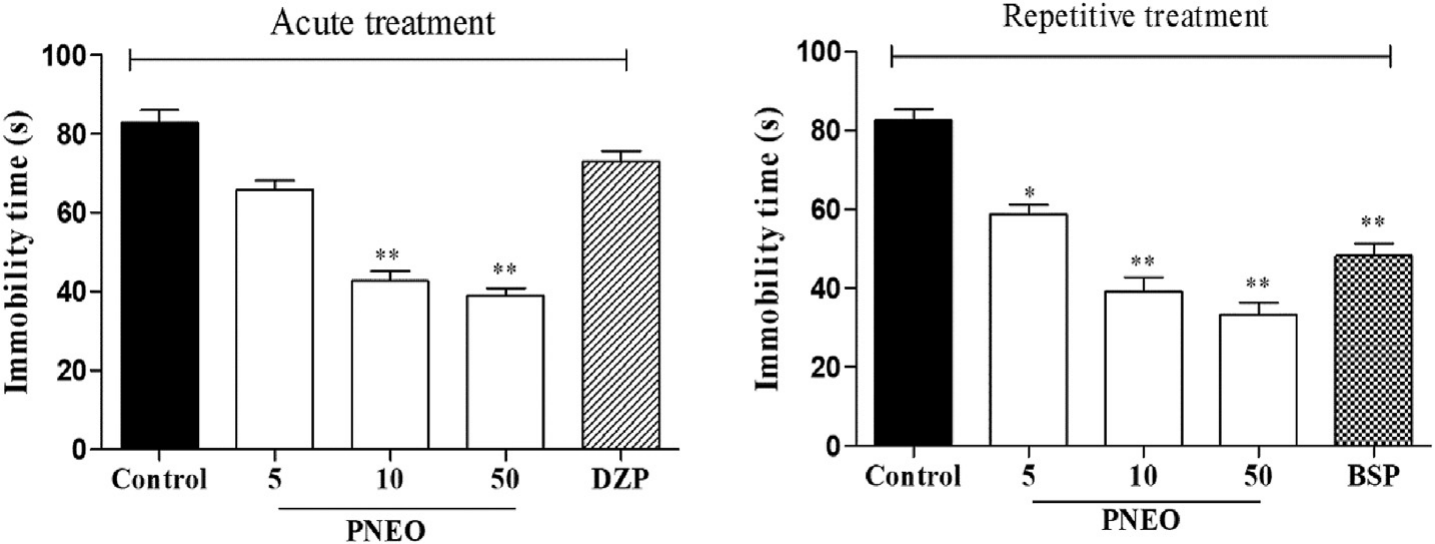
The effect of acute or repetitive treatments essential oil of *P. nigrum* (PNEO 5, 10 and 50 mg/kg, p.o.) on immobility time in the tail suspension test (TST). DZP: diazepam, 1 mg/kg (i.p.); BSP: buspirone, 10 mg/kg (i.p.). Data are represented as mean ± SEM (n = 6) and the values obtained by comparison with the control group using One-way ANOVA followed by Dunnett's test, where ∗ and ∗∗ indicates *p < 0.05* and *p < 0.01* respectively.

The results of the TST after the acute and repeated administration of the PNEO are represented in Figure 4. By comparing the vehicle group to the PNEO 10 and PNEO 50 group, there was a significant reduction in immobility time after acute administration. Whereas, a single dose of PNEO 5 and diazepam did not modify the immobility time in TST (Figure 4, left panel). The long term administration of PNEO at all doses and buspirone resulted in a significant reduction in the immobility time (Figure 4, right panel).

### 3.3 Mechanism(s) of action involved in the anxiolytic and antidepressant-like effect of PNEO

**Figure 5:**
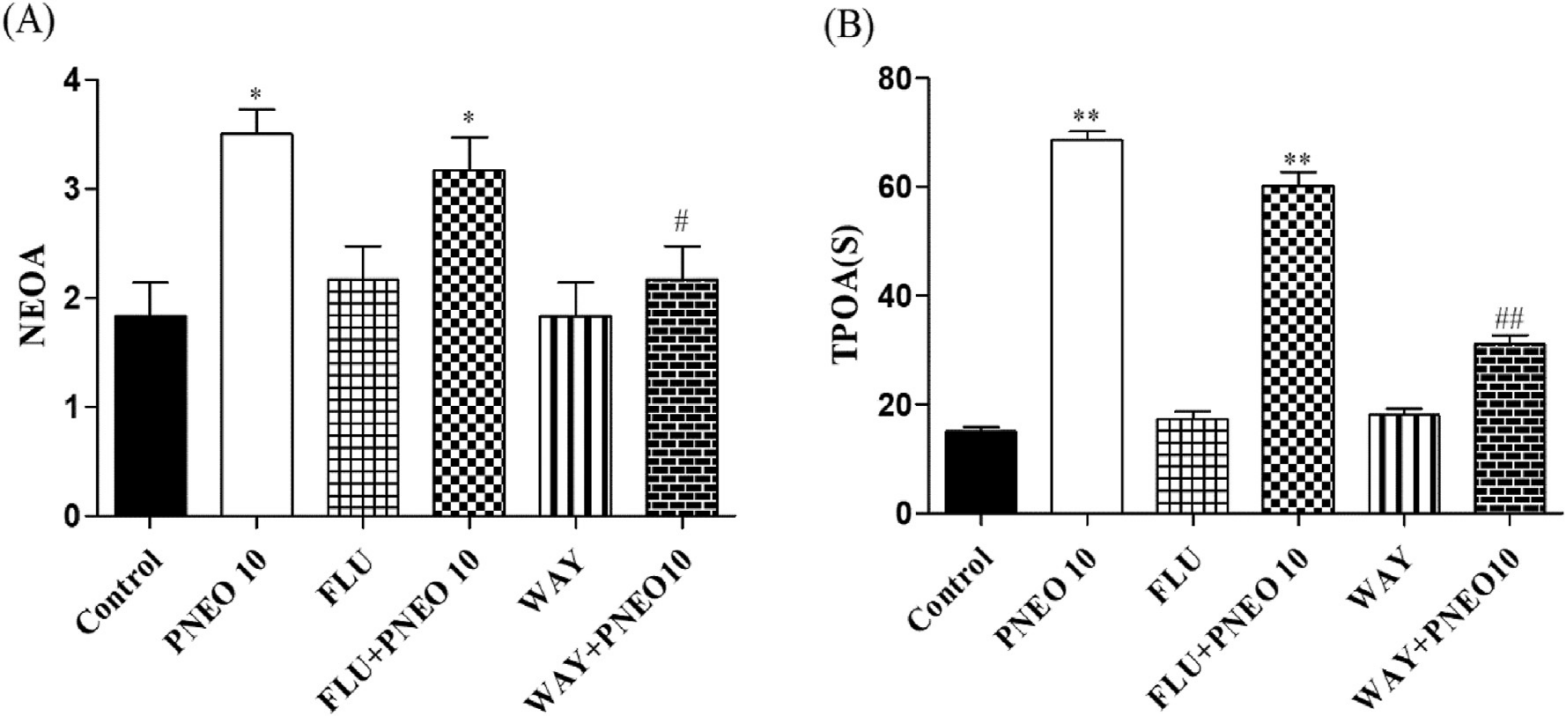
Effect of pre-treatment with flumazenil (FLU, 3 mg/kg, i.p.) and WAY-100635 (WAY, 1 mg/kg, i.p.) on the effect of the essential oil of *P. nigrum* (PNEO 10 mg/kg, p.o.) in the Elevated Plus Maze (EPM) test. (A) NEOA: Number of Entries in the Open Arms; TPOA: Time of Permanence in the Open Arms Data are represented as mean ± SEM (n = 6) and the values obtained by using One-way ANOVA followed by Dunnett's test, where ∗*p < 0.05, ∗∗p < 0.01* compared with the control group and ^*#*^*p < 0.05,*^*##*^*p < 0.01* compared with the PNEO (10 mg/kg, p.o.) treated group.

**Figure 6:**
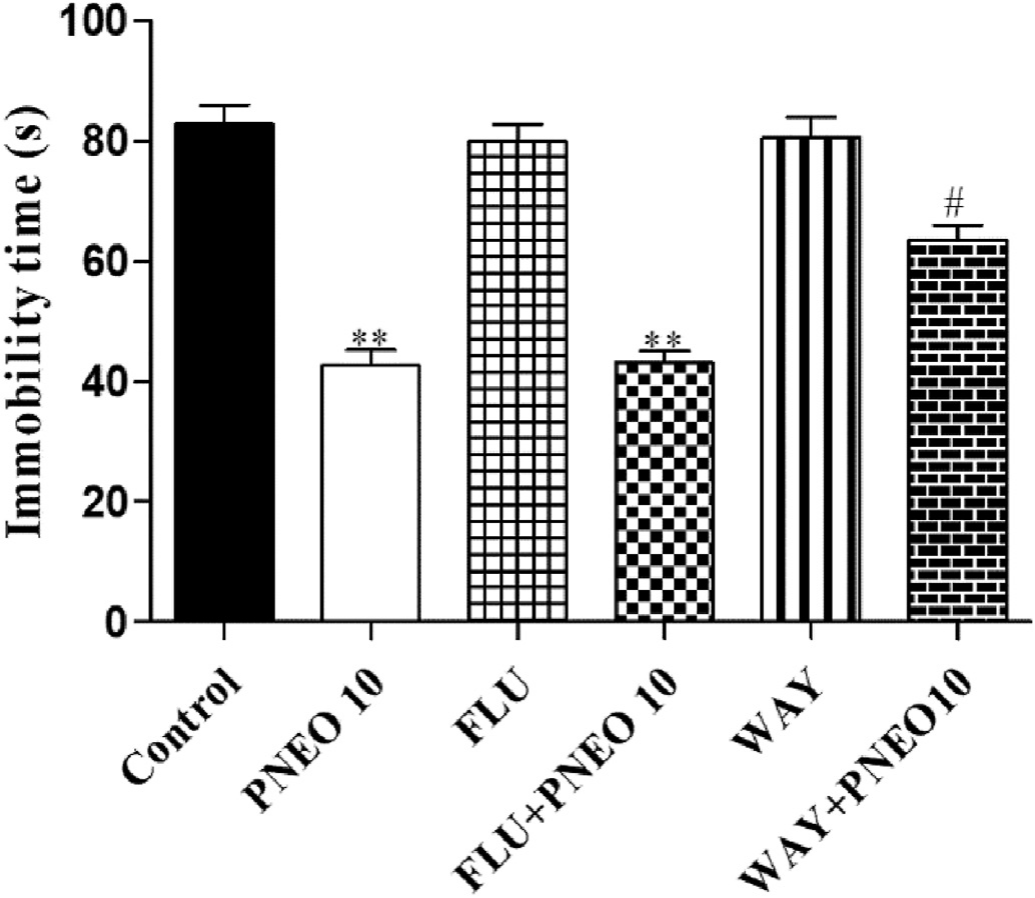
Effect of pre-treatment with flumazenil (FLU, 3 mg/kg, i.p.) and WAY-100635 (WAY, 1 mg/kg, i.p.) on the effect of the essential oil of *P. nigrum* (PNEO 10 mg/kg, p.o.) in the tail suspension test (TST). Data are represented as mean ± SEM (n = 6) and the values obtained by using One-way ANOVA followed by Dunnett's test, where. ∗∗*p < 0.01* compared with the control group ^*##*^*p < 0.01* compared with the PNEO (10 mg/kg p.o.) treated group.

To investigate the anxiolytic and antidepressant-like effect of PNEO showed in the EPM and TST respectively, is due to the involvement of GABA-ergic or serotonergic system, before receiving the single dose of PNEO10 (The dose was selected according to the findings of the series of the animal activity) were pre-treated with flumazenil or WAY-100635. As represented in Figure 5(A and B), pre-treatment with FLU could not block the effects of PNEO in EPM. However, pre-treatment with WAY significantly reversed the anxiolytic effect of PNEO. In the same way, pre-treatment with WAY but not FLU significantly reversed the effects of PNEO on the immobility time in the TST (Figure 6).

### 3.4 Neurochemical evaluation

Acute treatment with PNEO at all doses did not alter the level of monoamines or their metabolites (dopamine, 3, 4-dihydroxyphenylacetic acid, homovanillic acid, serotonin, and 5-hydroxy indole acetic acid) in the mice brain significantly (Table 2).

**Table 2:** Monoamine levels and their metabolites (ng/g) after acute oral treatment with PNEO.

	Control	PNEO (5 mg/kg)	PNEO (10 mg/kg)	PNEO (50 mg/kg)
DA	158.6 ± 16.4	148.5 ± 15.6	150.6 ± 18.2	143.8 ± 25.2
DOPAC	216.5 ± 24.6	220.8 ± 25.5	214 ± 26.6	210.4 ± 29.6
HVA	322 ± 32.8	308.6 ± 32.8	302.5 ± 30.8	299 ± 32.6
5-HT	184.4 ± 22.5	170.3 ± 18.6	162 ± 14.8	158 ± 22.4
5-HIAA	145.8 ± 16.3	139 ± 16.5	136.5 ± 18.2	132.8 ± 20.6

The animal were treated with vehicle (control), PNEO (5, 10 and 50 mg/kg, p.o.). Data are represented as mean ± SEM (n = 6) and the values obtained by comparison with control group using one-way ANOVA followed by Dunnett's test. DA- Dopamine; DOPAC-3, 4-dihydroxyphenylacetic acid; HVA- Homovanillic acid; 5-HT- Serotonin; 5-HIAA-5-hydroxyindoleacetic acid.

### 3.5 Toxicity and biochemical analyses

As shown in Tables (3 and 4), the 21-day PNEO treatment at all doses did not produce any noticeable sign of toxicity, changes in body weight or abnormalities in the biochemical parameters such as AST, ALP, ALT, glucose, urea, creatinine and triglycerides. The only parameter, total cholesterol was significantly reduced by the PNEO (10 and 50 mg/kg) treatment.

**Table 3:** Effects of repetitive treatments with PNEO on biochemical parameters.

Parameter	Treatment			
	Control	PNEO		
		(5 mg/kg)	(10 mg/kg)	(50 mg/kg)
AST (U/L)	94.6 ± 5.5	88.5 ± 4.4	101.3 ± 6.7	98.2 ± 6.3
ALP (U/L)	64.5 ± 6.1	72.2 ± 7.6	64.3 ± 4.5	75.6 ± 5.5
ALT(U/L)	51.4 ± 3.4	54.4 ± 6.1	58.6 ± 6.4	55.5 ± 4.2
Glucose (mg/dl)	254 ± 16.3	234.5 ± 14.7	221.6 ± 15.2	232 ± 15.6
Urea (mg/dl)	42.6 ± 4.7	48.2 ± 5.1	45.6 ± 6.2	40.8 ± 5.5
Creatinine (mg/dl)	0.56 ± 0.08	0.51 ± 0.04	0.48 ± 0.06	0.52 ± 0.04
Triglycerides (mg/dl)	94.6 ± 6.4	96.8 ± 4.4	76.5 ± 5.4	72.2 ± 3.8
Total cholesterol (mg/dl)	78.5 ± 5.5	66.64 ± 2.4	49.3 ± 3.8∗	44.6 ± 4.6∗

The animal were treated with vehicle (control), PNEO (5, 10 and 50 mg/kg, p.o.). Data are represented as mean ± SEM (n = 6) and the values obtained by comparison with control group using one-way ANOVA followed by Dunnett's test, Where ∗ indicates *p < 0.05*.

**Table 4:** Effects of repetitive treatments with PNEO on body weights.

	Day-1	Day-7	Day-14	Day-21
Control	26.4 ± 2.3	29.6 ± 3.1	32.6 ± 2.9	35.2 ± 2.7
PNEO (5 mg/kg)	25.8 ± 2.1	28.2 ± 2.9	30.6 ± 2.2	33.5 ± 2.5
PNEO (10 mg/kg)	26 ± 2.5	28.4 ± 3.2	29.3 ± 3.8	32.6 ± 2.9
PNEO (50 mg/kg)	26.5 ± 2.8	29.2 ± 2.5	31.8 ± 3.1	34.5 ± 2.8

The animal were treated with vehicle (control), PNEO (5, 10 and 50 mg/kg, p.o.). Data are represented as mean ± SEM (n = 6) and the values obtained by comparison with control group using one-way ANOVA followed by Dunnett's test.

## 4 Discussion

Plant EOs has always a prominent role in various traditional medicinal systems and is in use for centuries. Currently, researchers are keen to unfold their potential biological and therapeutic effects along with their possible association in the modern medicine system (Abouhosseini Tabari et al., 2018). Various compounds from different EOs have been identified as active biomolecules to affect conditions like anxiety, depression, and epilepsy due to their action upon the CNS (Rakotosaona et al., 2017). Despite the potential use of EOs from piper species in neurological disorders, no previous scientific reports on the neuropharmacological effect of *Piper nigrum* EO.

The major components, limonene, sabinene and caryophyllene detected from the PNEO was consistent with that reported by Bagheri (Bagheri et al., 2014). All of these terpenoids have been shown to have pharmacological effect on CNS, as shown by the decrease in anxiety and depression like symptoms in animals treated with these components.

(Koyama and Heinbockel, 2020). This study aimed to establish the anxiolytic and antidepressant-like effect of essential oils obtained from fruits of *Piper nigrum* through open field, elevated plus maze and tail suspension test.

Some classes of drugs like psychoactive stimulants give false-positive results due to the enhancement of the locomotor activity (Zhang et al., 2019). In our study, the spontaneous locomotor activity of mice and the effects of PNEO were investigated by the open field test. The findings revealed that acute and repeated PNEO administration has anxiolytic and antidepressant-like effects in mice without affecting locomotor function. The results show quite a good congruence with those described by Chioca, who suggested that linalool and linalyl acetate which are the main constituents of lavender EO exhibits anxiolytic effects in mice without modifying spontaneous locomotor activity (Chioca et al., 2013). A previous report indicated that CNS excitation is related to the rearing function (de Almeida et al., 2012). The central effects of PNEO (acute and repetitive treatment) are marked by a significant reduction of rearing and grooming behaviors in mice.

For the evaluation of the anxiolytic effect of PNEO, the elevated plus-maze test has been used which is based on the natural fear of rats and mice of height and wide-open spaces. In this model, the experimental animals avoided the open arms of the maze due to their natural fear of elevated zones (Lister, 1987). Also, the choice of this model as an anxiety test is due to its etiological validity. The number of entries and the permanence time in the open arms were taken as a marker to conclude the anxiolytic effect of the essential oil (Pellow et al., 1985). Our results show that there is an increase in the number of open arm entries and time of permanence in the open arm after the acute and repetitive administration of PNEO which is quite similar as in the case of diazepam and buspirone induced group. Several other EOs, like *P. roseum, C. citrates, Citrus limon* (Burn), *Spiranthera odoratissima* A. St. Hil., and *L. angustifolia* were reported to have similar findings (Abouhosseini Tabari et al., 2018; Blanco et al., 2009; Chioca et al., 2013; Galdino et al., 2012; Lopes C et al., 2011). Treatment of mice with *S. odoratissima* EO (500 mg/kg) and β-caryophyllene (100 and 200 mg/kg) resulted in an increase number of entries and permanence time in the open arms in the EPM test (Galdino et al., 2012).

The tail suspension test has certain benefits over the forced swimming test in that it allows for an objective measurement of immobility time and does not induce hypothermia by immersion in water (Cryan et al., 2005). Therefore, to evaluate the antidepressant activity in mice, tail suspension method was performed. Antidepressant drugs increased time (Kwon et al., 2010). In the TST, acute and repeated PNEO treatment decreased immobility time in a dose dependent manner, indicating that PNEO can be a choice of agent, which is used to manage depression. In agreement with our findings, EO from *Toona ciliate* Roem. Var. yunnanesis decreased the immobility time in TST and FST both (Duan et al., 2015).

Findings from our study show that PNEO exhibit both anxiolytic and antidepressant activity. Compounds having anxiolytic and antidepressant activity together is extremely useful and imparts a better therapeutic response particularly in the treatment of comorbid anxiety and depression-like symptoms. Anxiety and depression are often associated together and seen in some patients. An estimated 90% of patients with anxiety disorders still have depressive symptoms. Patients who have both depression and anxiety have more serious symptoms and a poor treatment response than patients who only have one disorder (Abouhosseini Tabari et al., 2018; Gorman, 1996). These facts can enlighten the effectiveness of compounds that can treat anxiety and depression at the same time. Therefore, the PNEO can be considered as a natural remedy for attenuating symptoms of comorbid anxiety and depression.

Subsequently, we decided to investigate the possible underlying mechanism of PNEO. In the EPM, pre-treatment with WAY 100635, although not flumazenil, significantly reversed the anxiolytic-like activity (number of entries and permanence time in the open arm) of PNEO. The immobility time of the PNEO treated group in TST was also significantly reduced after pre-treatment with WAY 100635. These findings might indicate that the anxiolytic and antidepressant-like effects of PNEO are likely mediated by the serotonergic transmission system. Similar findings have been reported from *P. roseum* EO, which exhibited their anxiolytic and antidepressant effects via 5-HT receptor (Abouhosseini Tabari et al., 2018). However, we cannot discard the prospect that the compounds from PNEO interact with other monoamine systems. In anxiety disorders, the central dopaminergic system is considered one of the most important factors and a previous report suggested that most neuropharmacological effects through dopamine transmission directly controlled by serotonin transmission system (Scalzitti et al., 1999). Furthermore, studies on limonene showed that it inhibits methamphetamine-induced locomotor activity possibly through regulation of 5-HT and dopamine neurotransmission (Yun, 2014). Therefore, we further decided to perform the neurochemical analysis and the neurochemical assessment revealed no alterations in the levels of DA, 5HT and their metabolites in the brain after acute treatments with the PNEO. It has been indicated that dopamine transmission could not involve in the effect of PNEO, but still long-term treatment will be required to reveal its effect on neurotransmission systems.

In agreement with the research guidelines for evaluating the safety and efficacy of herbal medicines decided by WHO in 1993, the 21-days repeated PNEO treatment in this study produced no adverse events. Regular inspection and biochemical study represented no marked alteration in body weight or the biochemical parameters of the treated animals, except for a reduction in total cholesterol level. In line with this finding, a study reported that a major compound, limonene containing lemon essential oil lowers the cholesterol level in hypercholesterolemia-induced rabbits (Lee et al., 2018).

Finally, essential oils are compound mixtures of diverse molecules. The major compounds of the EO from *Piper nigrum* were identified as limonene. According to a previous report, limonene was unable to alter the parameters of anxiolytic-like activity evaluated in the EPM test (do Vale et al., 2002). Furthermore, a previous study reported that a major identified compound, limonene from *Citrus aurantium* was not able to modify the immobility time (Costa et al., 2013). These shreds of evidence indicate that anxiolytic and antidepressant-like effects of PNEO cannot be attributed to the major compound, limonene only but also the combined synergistic effects of components present in it. According to the theory of a multi-targeted approach as a therapeutic strategy, only one constituent from the herbal preparation cannot be always responsible for every bioactivity (Wagner and Ulrich-Merzenich, 2009). Moreover, for therapeutic purposes, it is more explanatory to study crude essential oil rather than some of its compounds because the hypothesis of synergism appears to be more significant (Bakkali et al., 2008).

## 5 Conclusion

*Piper nigrum* EO possesses a significant anxiolytic and antidepressant-like effect through the possible involvement of serotonergic transmission. Also, no signs of toxicity were produced by EO at different doses and thus appeared to be well tolerated. Our findings highlight that lipophilic volatile compounds from natural products can be a good source for discovering new medicinal agents having a fast onset of action and less adverse effects for the treatment of comorbid anxiety and depression. However further studies are required to compare the whole EO of *P. nigrum* and its major constituents for the neuropharmacological effects and their possible mechanisms of action and discover meaningful synergisms between them.

## Declarations

### Author contribution statement

Sourav Ghosh^a^: Conceived and designed the experiments; Performed the experiments; Contributed reagents and wrote the paper.

Arvind Kumar^b^ and Neetu Sachana^a^: Analyzed and interpreted the data; Contributed reagents, materials, analysis tools or data.

Phool Chandra^a^: Conceived and designed the experiments; Performed the experiments; wrote the paper.

### Funding statement

This research did not receive any specific grant from funding agencies in the public, commercial, or not-for-profit sectors.

### Data availability statement

Data included in article/supplementary material/referenced in article.

### Declaration of interests statement

The authors declare no conflict of interest.

### Additional information

No additional information is available for this paper.
